# Screening method toward ClbP-specific inhibitors

**DOI:** 10.1186/s41021-023-00264-7

**Published:** 2023-02-16

**Authors:** Tao Zhou, Takayuki Ando, Akihiro Kudo, Michio Sato, Noriyuki Miyoshi, Michihiro Mutoh, Hideki Ishikawa, Keiji Wakabayashi, Kenji Watanabe

**Affiliations:** 1 Adenoprevent Co., Ltd., Shizuoka, 422-8526 Japan; 2 Department of Pharmaceutical Sciences, University of Shizuoka, Shizuoka, 422-8526 Japan; 3 Department of Pharmaceutical and Food Science, Shizuoka Institution of Environment and Hygiene, Fujieda, 426-0083 Japan; 4 Graduate School of Integrated Pharmaceutical and Nutritional Sciences, University of Shizuoka, Shizuoka, 422-8526 Japan; 5 Department of Molecular-Targeting Cancer Prevention, Kyoto Prefectural University of Medicine, Kyoto, 602-8566 Japan; 6 Graduate School of Nutritional and Environmental Sciences, University of Shizuoka, Shizuoka, 422-8526 Japan

**Keywords:** Colibactin, ClbP, Inhibitor, Screening, Fluorescent, Probe

## Abstract

**Background:**

Colibactin is a genotoxin produced by *Escherichia coli* and other *Enterobacteriaceae* that is believed to increase the risk of colorectal cancer (CRC) of their symbiosis hosts, including human. A peptidase ClbP is the key enzyme for activation of colibactin. Inhibition of ClbP is considered to impede maturation of precolibactin into genotoxic colibactin. Therefore, ClbP-specific inhibitors could potentially prevent the onset of CRC, one of the leading causes of cancer-related deaths in the world. This study intends to establish an efficient screening system for identifying inhibitors that are specific to ClbP.

**Methods:**

Two types of assays were applied in the screening procedure: a probe assay and an LC–MS assay. For the probe assay, we employed the synthesized probe which we described in our previous report. This probe can be hydrolyzed efficiently by ClbP to release a fluorophore. Hence it was applied here for detection of inhibition of ClbP. For the LC–MS assay, formation of the byproduct of precolibactin maturation process, *N*-myristoyl-D-asparagine, was quantified using a liquid chromatography–mass spectrometry (LC–MS) technique. The probe assay can be performed much faster, while the LC–MS assay is more accurate. Therefore, our method employed the two assays in sequence to screen a large number of compounds for inhibition of ClbP.

**Results:**

A library of 67,965 standard compounds was evaluated by the screening method established in the current study, and one compound was found to show a moderate inhibitory activity against ClbP.

**Conclusion:**

A simple screening method for ClbP-specific inhibitors was established. It was proven to be reliable and is believed to be useful in developing potential prophylactic agents for CRC.

## Introduction

Colibactin is a genotoxin secreted by bacteria belonging to the family *Enterobacteriaceae*. It is biosynthesized via a PKS–NRPS hybrid pathway encoded within a 54-kilobase genomic island called *pks* or *clb* [1]. The causal relationship between colibactin and colorectal cancer (CRC) was inferred by statistical analysis and in vitro and in vivo experiments [1–3]. Since CRC is one of the leading causes of cancer-related deaths in the world [4], there is a strong interest in understanding the mechanism of carcinogenesis induced by colibactin. As such, much effort went into elucidating the chemical structure and biosynthetic pathway of colibactin over the past decade [5]. However, because colibactin is structurally unstable, it eluded successful isolation and structural determination for a long time. Initial attempts at determining the chemical structure of colibactin relied on proposed structures based on the biosynthetic analysis of the enzymes encoded by the genes in the *clb* island. In addition, structural information obtained from colibactin precursors, which are immature forms of colibactin generated by mutant strains having the *clb* genes knocked out, and stable colibactin derivatives formed after spontaneous or artificial transformations of the intact colibactin [6, 7]. Structural studies revealed that colibactin contains a cyclopropane moiety that is considered to react with DNA to cause alkylation and cross-linking of the DNA molecules [8]. Such chemical alterations lead to single- and double-stranded breakage of DNA that can induce carcinogenesis of cells [9].

**Fig. 1:**
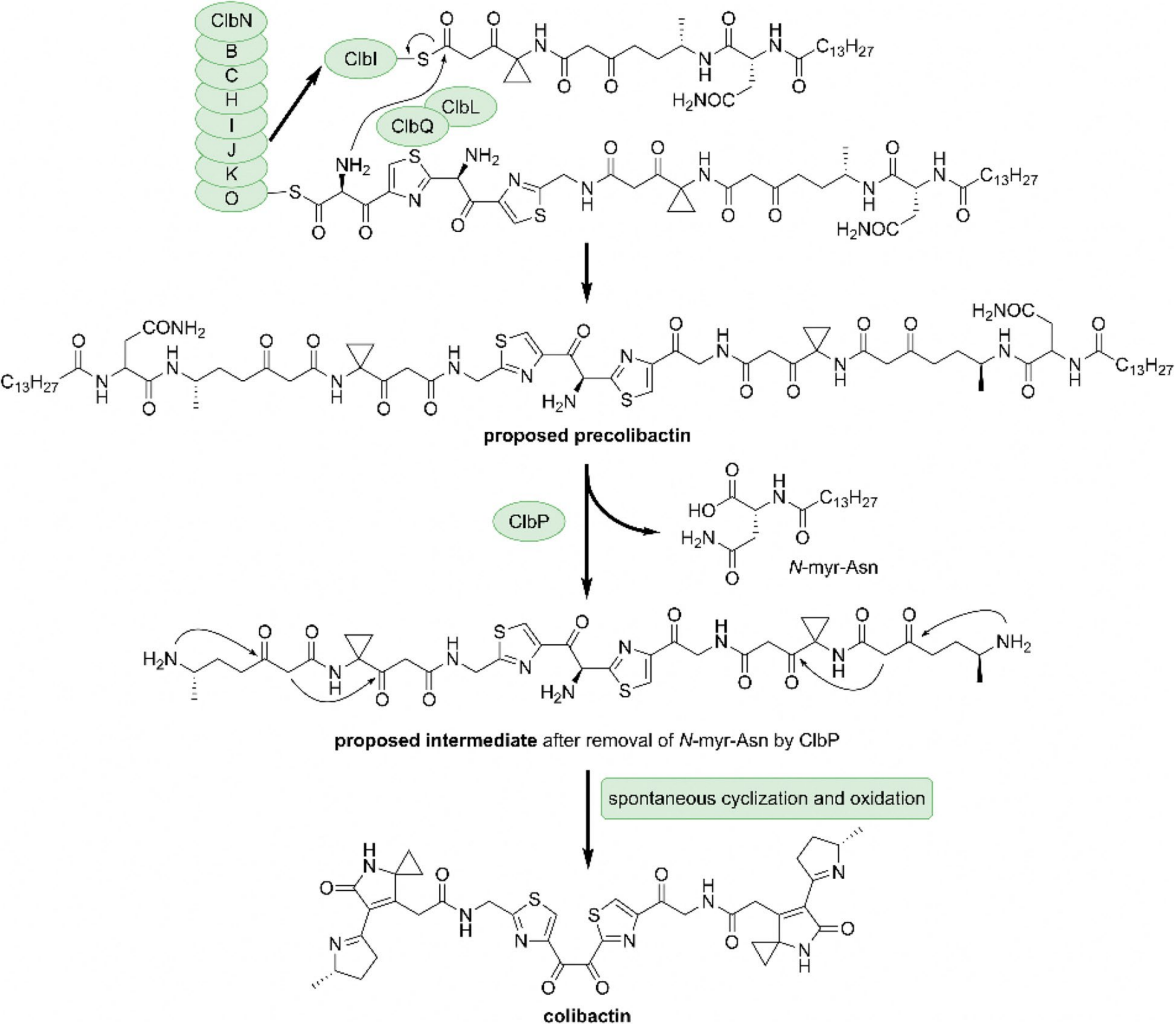
Proposed biosynthetic pathway of colibactin. *N*-myr-Asn: *N*-myristoyl-D-asparagine

Biosynthetic pathway of colibactin was proposed as shown in Fig. 1. The precursor of colibactin (Fig. 1, proposed precolibactin) is first synthesized by the actions of multiple polyketide synthases (PKSs), nonribosomal peptide synthetases (NRPSs), NRPS–PKS hybrid enzymes and several other additional enzymes (Fig. 1, ClbB,C,H,I,J,K,L,N,O,Q) [9]. Precolibactin is activated into a plausible reactive intermediate (Fig. 1, proposed intermediate) by the removal of the terminal *N*-myristoyl-D-asparagine (*N*-myr-Asn) moiety by the peptidase ClbP in a way similar to how a prodrug is activated by the removal of a protecting group [10]. The presumed mature colibactin is thereafter generated following spontaneous intramolecular cyclization and oxidation reactions (Fig. 1, colibactin). Being the final catalyst in this pathway, ClbP plays an important role in generating the mature structure of colibactin as well as implementing its reactivity toward DNA. This catalytic process is unique and considered as a standard for identifying colibactin producing (*clb*+) strains.

In our previous report, activity-based fluorogenic probes mimicking the substrate of ClbP were designed and synthesized to identify quickly and easily the presence of colibactin-producing bacteria in fecal samples of CRC patients [11]. Presently, this technique is commercially applied to evaluate the CRC risk of subjects [12]. As evidenced by the accumulating statistical data, a high proportion of CRC patients was expectedly found to host the *clb* + strains. However, astonishingly an average of approximately 20% of subjects within the control group, namely healthy individuals, were also *clb*+, suggesting that infection by the *clb* + strains is spread widely across a large region, including North America, Europe, and East Asia [13–17]. Moreover, our recent study indicated that *clb* + strains can be transmitted from mother to infant through intimate contacts such as natural childbirth and breastfeeding [18]. Currently most of *clb* + individuals are unaware of their exposure to colibactin-producing pathogens, and consequently no therapy has been developed to control such infections. Previous studies have shown that ClbP plays a. crucial role in the generation of the genotoxically active form of colibactin [10] and knocking out *clbP* leads to generation of a mutant showing no cytopathic effect [1]. Therefore, screening for specific inhibitors of ClbP would be a viable approach in developing therapeutics that can suppress the production of colibactin, which in turn could potentially lower the risk of CRC within *clb +* individuals while minimizing the negative side effects that may be brought about by antibiotic treatments for eradicating enteric *clb +* strains. In this paper, we present an in vivo screening method aiming at finding specific inhibitors of ClbP by applying the activity-based probe we have developed in combination with an LC–MS assay to improve both the efficiency and accuracy of the screening method.

## Materials and methods

### Strains, culture conditions, chemicals, and instruments

*E. coli*-50 isolated from a human CRC tissue that was described in our previous report [11] was employed as the reference colibactin-producing strain and its Δ*clbP* mutant as a negative control strain. All bacteria were cultured in EC broth (Nissui Pharmaceutical Co., Ltd.) at 37 °C with a starting concentration of 10^5^ colony forming unit (cfu). A library of commercially available 67,965 compounds owned by Fuji Pharma Valley Center (Nagaizumi, Shizuoka), was provided by Shizuoka Institution of Environment and Hygiene for the screening [19]. The florescent probe for the probe assay, *N*-myristoyl-D-asparaginyl-4-methylcoumarin, was synthesized following our previous report [8]. Fluorescent intensity was record with a Molecular Devices Spectramax Gemini EM microplate reader. LC–MS spectra were recorded with a Thermo Fisher Scientific Q-Exactive liquid chromatography–mass spectrometer (LC–MS) using both positive and negative electrospray ionization (ESI) modes. Samples were separated for analysis on an ACQUITY UPLC 1.8 μm, 2.1 × 50 mm C18 reversed-phase column (Waters) using a linear gradient of 5–50% (v/v) MeCN in H_2_O supplemented with 0.05% (v/v) formic acid at a flow rate of 0.5 mL/min.

### Probe assay

**Fig. 2:**
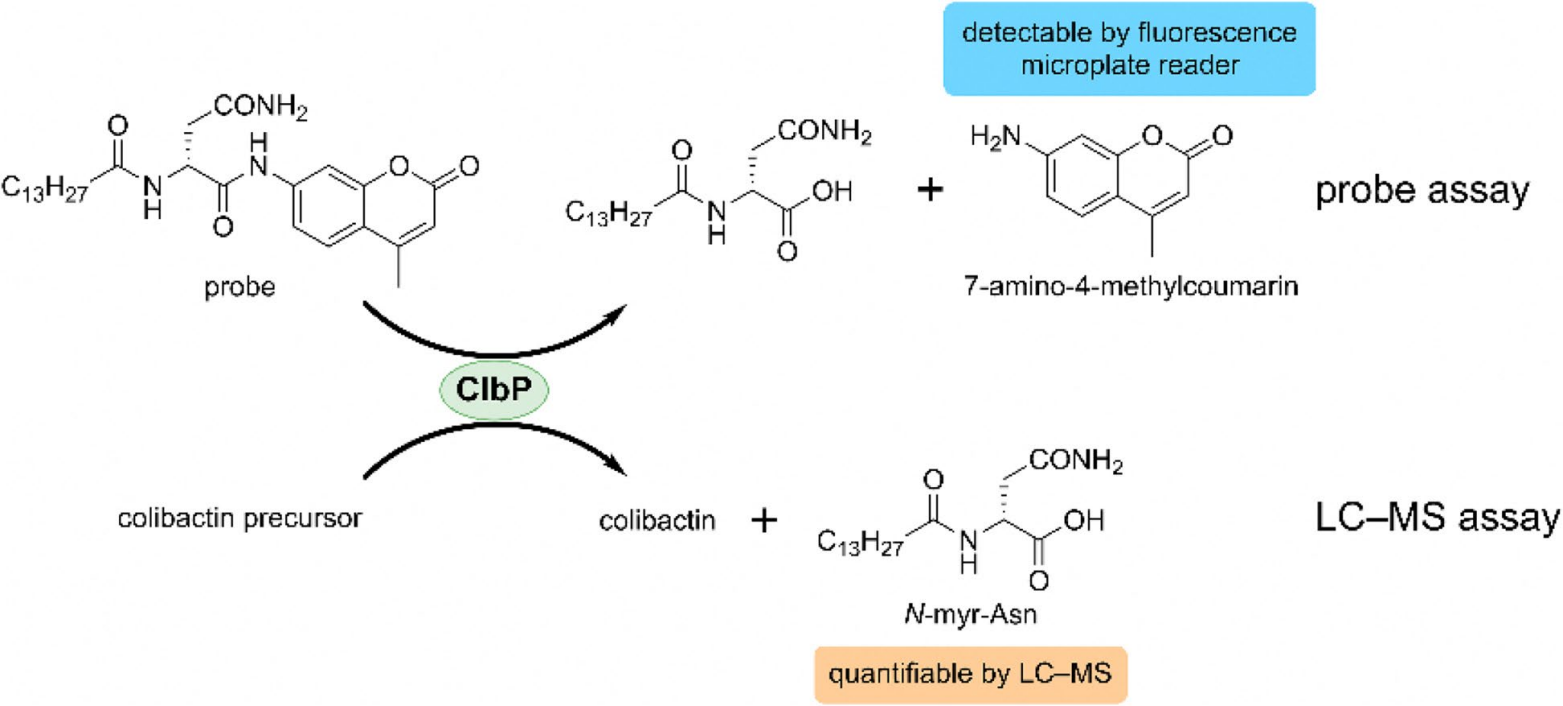
Mechanisms of the probe and LC–MS assays employed in the current study

The fluorescent probe (Fig. 2) was designed to be effectively hydrolyzed by ClbP and release 7-amino-4-methylcoumarin, a fluorescently detectable moiety [11]. Therefore, when a *clb +* bacteria producing ClbP is cultured in the presence of this probe, the culture would give a strong fluorescence signal. On the other hand, only a weak fluorescent signal would be detected if ClbP was inhibited. Based on this mechanism, we designed the screening protocol as described below. The synthesized and purified fluorescent probe was dissolved in dimethylsulfoxide (DMSO) at the concentration of 20 mM as a stock probe solution. The seed cultures of the *E. coli*-50 and *E. coli*-50/Δ*clbP* strains were prepared in EC broth and adjusted to a concentration of 10^5^ cfu. The stock probe solution was added to the concentration-adjusted cultures to the final concentration of 1 mM. The compounds being tested as inhibitors were dissolved and stored in DMSO before use. To black 96-well microplates with clear bottom, 98 μL of the probe–culture mix and 2 μL of each of the test compound solutions were added to each well. For the positive and negative controls, the probe–*E. coli*-50 culture mix and probe–*E. coli*-50/Δ*clbP* culture mix were added with 2 μL DMSO only instead of the test compound solution. The plates were sealed and incubated at 37 °C for 72 hours. The fluorescent signal with excitation and emission wavelengths of 350 and 460 nm, respectively, was recorded. The inhibition rate was calculated according to the following equation:

$$Inhibition\ Rate=\left(1-\frac{\varDelta_{Sample}-{\varDelta}_{negative}}{\varDelta_{positive}-{\varDelta}_{negative}}\right)\times 100\%$$

where Δ refers to the increase in the fluorescent intensity after incubation.

### LC–MS assay

The LC–MS assay for quantification of *N*-myr-Asn in the culture sample was carried out in the following manner.

The probe–culture–compound mixtures including the positive and negative controls were prepared in black 96-well microplates as described above. The plates were sealed and incubated at 37 °C for 24 hours. After cultivation, 80 μL of the mixture was removed from the well to a microtube and mixed with 8 μL of an inner standard solution (*N*-myr-Asn-*d*_27_ in DMF at 500 ng/mL) and 712 μL diluted hydrochloric acid (1 N). The solution was added with 800 μL EtOAc. After vortexing and centrifuging, 500 μL of the organic layer was removed, and the solvent was evaporated. The residue was dissolved in 50 μL dimethylformamide (DMF) and analyzed by LC–MS. The integration areas of *N*-myr-Asn and *N*-myr-Asn-*d*_27_ were acquired from each corresponding SIC spectra and used in the following equation to calculate the inhibition rate:

$$Inhibition\ Rate=\left[1-\left(\frac{Area_{H- sample}}{Area_{D- sample}}-\frac{Area_{H- negative}}{Area_{D- negative}}\right)/\left(\frac{Area_{H- positive}}{Area_{D- positive}}-\frac{Area_{H- negative}}{Area_{D- negative}}\right)\right]\times 100\%$$

where *Area*_*H*_ and *Area*_*D*_ refer to the integration areas of *N*-myr-Asn and *N*-myr-Asn-*d*_27_, respectively.

## Results and discussion

As briefly described earlier, two screening methods were designed for the current study. The probe assay was based on the use of our activity-based fluorogenic probe developed against ClbP, while the LC–MS assay exploited the available LC–MS technology to quantitate the byproduct *N*-myr-Asn produced by ClbP. The concepts of the two screening methods are illustrated in Fig. 2. Colibactin is well known to be structurally unstable and extremely difficult to isolate, let alone quantify. Thus, production of colibactin must be detected in ways other than measuring the production of colibactin itself. Therefore, we developed a fluorogenic probe that is specifically activated by ClbP [11]. Because the hydrolytic activity of ClbP is directly coupled with generation of the mature form of colibactin, sensitive in vivo detection of the ClbP activity proved to be an effective approach in determining the level of colibactin formation in *clb* + strains [11]. Performing the screening assay using the actual live *E. coli* cells also reduces the likelihood of identifying compounds that are ineffective in vivo. Similarly, *N*-myr-Asn is much more stable and quantifiable than colibactin. Since *N*-myr-Asn is uniquely generated during the catabolism for mature colibactin production, it can also serve as an excellent marker for colibactin production. Moreover, because removal of *N*-myr-Asn from precolibactin requires processing by ClbP (Fig. 1), it also serves as a direct indicator of ClbP activity. Thus, we can exploit the high resolution of currently available LC–MS technology to quantitative assess the activity of ClbP by measuring the quantity of *N*-myr-Asn generated in a sample. To standardize the readouts from the LC–MS instrument, *N*-myr-Asn-*d*_27_ was synthesized and included in the LC–MS analysis as an internal standard. Synthesis of *N*-myr-Asn-*d*_27_ was accomplished by essentially the same protocol we developed previously for the synthesis of *N*-myr-Asn [11] except that myristic acid-*d*_27_ instead of unlabeled myristic acid was used as a starting material.

The two methods employed in this study have their pros and cons that can complement each other. The probe assay is simple and sensitive and can handle multiple samples in parallel. On the other hand, the LC–MS assay can analyze the samples only in a sequence, and each sample requires a fixed amount of time to be analyzed. Those differences bring much difference to their efficiency. However, since the probe assay is dependent on fluorescent signal readouts, interfering UV absorption or fluorescent excitation and emission by various components in the test samples might generate false positive or negative results. Such spectroscopic factors will not affect the LC–MS results. In consideration of both efficiency and accuracy of the screening workflow, the probe assay was employed for preliminary screening of large number of compounds, and the LC–MS assay was used in the subsequent confirmation of the activities of the candidates identified during the initial screening step.

A chemical library containing 67,965 compounds of small molecule with a focus on heterocyclic for drug discovery. For the first step of the screening, a library holding a total number of 67,965 compounds was screened by the probe assay. To speed up the workflow, ten compounds were pre-mixed as a single sample batch before the assay. To prepare a pre-mixed batch, an equal amount of each compound (10 mM in dimethyl sulfoxide) was mixed to yield a batch containing ten compounds, each at 1 mM. After 6952 batches of pre-mixed library compounds were prepared, each batch was added to a well in a black 96-well microplate, and the final concentration of each of the ten compounds in a well was adjusted to 20 μM. Upon addition of bacterial culture and the fluorogenic probe, each well was examined for the fluorescence emission signal at 460 nm. Screening of the plates identified 48 batches that showed > 80% inhibitory activity. Since the apparent inhibition could arise from not only specific ClbP inhibition but also antibiotic activities of the test compounds that suppressed bacterial growth, the turbidity of the sample mixture in each well was also examined by reading its optical density at 600 nm (OD600). Because the positive control showed an average OD600 of 0.58, the sample mixtures with OD600 less than 0.3 were considered to have been affected by the antibiotic activity of the test compounds. Thus, based on the OD600 readouts, 16 batches were removed from the candidate list, and remaining 32 batches or 320 test compounds were selected for further characterizations.

**Fig. 3:**
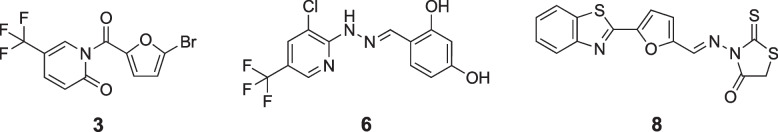
The structures of test compounds 3, 6, and 8

In the second step, the 320 test compounds selected during the first screening step were re-examined individually by the same protocol used during the first step. After removing six compounds with OD600 readouts below 0.3, 17 test compounds were found to be potential ClbP inhibitors. As the third step of the screening, those 17 compounds were examined further by the LC–MS assay. The results are shown in Table 1. Unfortunately, our screening efforts did not identify any potent ClbP inhibitor. Only a moderate inhibitory activity was observed with a few compounds, including compound 6 that showed 66% inhibition of the ClbP activity. The structures of the top three inhibitory compounds are shown in Fig. 3. It is not surprising to find that a large proportion of the test compounds identified by the probe assay showed poor activity, as interfering fluorescent signals can be emitted by various substances present in the sample mixture that can give rise to false positive results. Nevertheless, the LC–MS assay in the third step of screening was able to identify a selection of compounds with moderate inhibitory activity. Thus, the current result proved that the screening protocol we designed is efficient and reliable in identifying compounds that can inhibit the activity of ClbP. We believe that by expanding the screening range, more potent compounds can be discovered with this method.

**Table 1:** Inhibitory activity of the compounds examined in the third step of the screening

Compound	Inhibition rate
**1**	0%
**2**	32%
**3**	41%
**4**	20%
**5**	10%
**6**	66%
**7**	− 14%
**8**	50%
**9**	21%
**10**	−26%
**11**	2%
**12**	2%
**13**	3%
**14**	5%
**15**	39%
**16**	8%
**17**	4%

## Conclusions

In the current work, a screening method aiming at discovering specific ClbP inhibitors was established. The procedure was comprised of three steps that combined a probe-based assay and an LC–MS-based assay to ensure overall efficiency and accuracy. A chemical library containing 67,965 compounds was screened with this method, and a candidate compound 6 with a moderate ClbP inhibitory activity was identified. In the following research, we intend to expand the search space for identifying more potent inhibitors. We also plan to examine the mechanism of inhibition by the compounds identified through the screening exercise using computational, biochemical and structural methods. The outcome of this preliminary screening work attested to the reliability of the screening method we designed. The simple protocol employed in the current screening method also has the potential to be modified into an automated high-throughput screening method to further improve the search efficiency. The method we proposed here can serve as a starting point toward the discovery of compounds with potent ClbP inhibitory activities that can be developed into chemoprophylactic agents against CRC.

## Data Availability

The datasets generated and/or analyzed during the current study are available from the corresponding authors on reasonable request.
